# The role of carbon dioxide angiography in reducing contrast-induced nephropathy in diabetic foot patients undergoing endovascular treatment

**DOI:** 10.55730/1300-0144.6040

**Published:** 2025-05-30

**Authors:** Sadık Ahmet UYANIK, Erdem BİRGİ, Saffet ÖZTÜRK, Umut ASFUROĞLU, Erdi TANGOBAY, Hikmet Erhan GÜVEN

**Affiliations:** 1 Department of Radiology, Etlik City Hospital, Ankara, Turkiye; 2 Department of Radiology, Ankara Training and Research Hospital, University of Health Sciences, Ankara, Turkiye; 3 Department of General Surgery, Etlik City Hospital, Ankara, Turkiye

**Keywords:** Angiography, carbon dioxide, contrast media, diabetic foot, endovascular procedures, peripheral arterial disease

## Abstract

**Background/aim:**

This study investigates the role of carbon dioxide (CO_2_) angiography, delivered with an automated CO_2_ delivery system, in decreasing the amount of iodinated contrast and preventing contrast-induced nephropathy (CIN) in diabetic foot patients who underwent endovascular revascularization.

**Materials and methods:**

A total of 272 diabetic foot patients who underwent endovascular treatment for infrainguinal chronic peripheral arterial disease (PAD) were included in the study. Of these, 64 patients underwent endovascular intervention using CO_2_ angiography (study group), while 208 patients underwent endovascular intervention using only contrast media (control group). The rates of CIN and the amount of contrast used during interventions were recorded alongside secondary outcomes, including technical success, complication rates, and complications related to CO_2_ usage.

**Results:**

The mean contrast volume used in the CO_2_ group was significantly lower than in the control group (24.3 ± 13.3 cc vs 89.4 ± 24.8 cc; p < 0.001). CIN was detected in 41 patients. The incidence of CIN was 17.7% in the control group, while it was significantly lower in the CO_2_ group at 6.2% (p = 0.024). In a subgroup of patients with chronic kidney disease stage 3–5, CIN incidence remained significantly lower in the CO_2_ group (6.2% vs 38.2%, p < 0.001), and multivariate analysis identified CO_2_ use as an independent protective factor (OR: 0.027, 95% CI: 0.005–0.133, p < 0.001). Technical success rates were comparable between the groups (93.7% vs 93.2%; p = 0.892). Pain after CO_2_ injection was recorded in 11 patients, and no other adverse effect due to CO_2_ usage was observed. There were no major complications, and only minor complications occurred (8%).

**Conclusion:**

CO_2_ angiography may play a crucial role in minimizing the risk of CIN in this specific population, who are more vulnerable to this complication and its associated morbidity and mortality. Further multicenter prospective studies are needed to better define the role of CO_2_ angiography in high-risk patients.

## 1. Introduction

Diabetic foot is an aggressive complication of long-standing uncontrolled diabetes. Foot ulceration accompanied by infection commonly results in major amputation and increased morbidity and mortality [1]. Along with dedicated wound care, glycemic control, and antibiotherapy, revascularization plays a crucial role in the treatment of chronic limb-threatening ischemia and diabetic foot ulcers. As these patients often have multiple comorbidities and lack suitable by-pass options, endovascular treatment has become the first-line option, paralleling the improvements of current endovascular equipment and techniques.

The increasing number of endovascular interventions raises concerns regarding the amount of iodinated contrast used and the risk of contrast-induced nephropathy (CIN) associated with its use. CIN is a potential cause of increased morbidity and hospital stays; thus, preventing CIN is of paramount importance [2, 3]. Diabetic patients are particularly more prone to CIN due to preexisting glomerular nephropathy, the use of nephrotoxic drugs, and underlying microangiopathy [4]. Although multiple factors contribute to the development of CIN, the volume of iodinated contrast is considered an independent risk factor. The use of carbon dioxide (CO_2_) as a contrast medium during endovascular interventions represents a valuable alternative to iodine-based contrast. Unlike liquid iodine-based contrast medium, which dissolves in blood, gaseous CO_2_ displaces the blood. As the atomic number and density are low, CO_2_ acts as a negative contrast agent and requires high-quality digital subtraction imaging. With the development of safe delivery systems, CO_2_ angiography has become widely used in patients with renal impairment [5–9].

This study investigates the role of CO_2_ angiography, delivered with an automated CO_2_ delivery system, in decreasing the amount of iodinated contrast and preventing CIN in diabetic foot patients who underwent endovascular revascularization.

## 2. Materials and methods

### 2.1. Study population

This study was conducted in the interventional radiology unit of a large-volume tertiary healthcare center. Digital subtraction angiography images, procedure reports, clinical examination findings, and laboratory results of 492 patients, referred from the wound care unit and treated for lower extremity peripheral artery disease between June 2023 and December 2024, were retrospectively analyzed. The study was designed in accordance with the Declaration of Helsinki and approved by the institutional ethics committee (Approval date: 12.03.2025; decision number: 2025-349). Informed consent was obtained from all patients prior to the procedures.

Patients presenting with acute limb ischemia, nondiabetic patients, patients already receiving renal replacement therapy, patients treated solely for aortoiliac disease, patients whose treatment was terminated after diagnostic angiography, and patients with missing data were excluded.

**Figure 1:**
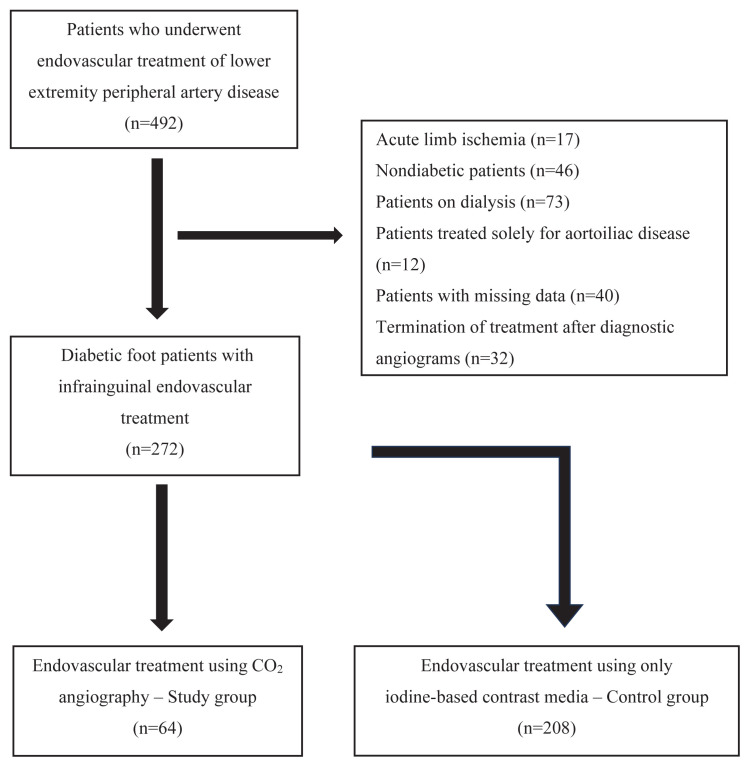
Flowchart of the study population and group allocation.

A total of 272 diabetic foot patients who underwent endovascular treatment for infrainguinal peripheral arterial disease were included in the study. Of these, 64 patients underwent endovascular intervention using CO_2_ angiography (study group), while 208 patients underwent endovascular intervention using only iodine-based contrast media (control group). The flowchart is presented in Figure 1.

The primary outcomes of the study were CIN and the amount of contrast used during interventions. Secondary outcomes included technical success, complication rates, and complications related to CO_2_ usage.

Serum creatinine levels were routinely measured preintervention and within 48–72 h postprocedure. CIN was defined as an absolute increase of ≥0.5 mg/dL or a ≥25% relative increase from baseline within this interval. All included patients had at least one follow-up creatinine measurement during hospitalization. Technical success was defined as successful recanalization of the treated artery with <30% residual stenosis and recanalization of at least one outflow artery to the foot.

The Cardiovascular and Interventional Radiological Society of Europe (CIRSE) classification system was used to assess the complications [10].

### 2.2. Endovascular intervention

All interventions were performed by a senior interventional radiologist with 10 years of experience. Under local anesthesia, retrograde common femoral artery or antegrade superficial femoral artery access was achieved, followed by diagnostic angiography. Angiographies were performed using the Innova IGS 630 (Ge Healthcare, Chicago, IL, USA) digital subtraction angiography system. After depiction of the target lesions, a support catheter and guidewire combination was used to cross the lesions. Based on lesion characteristics, 0.035-inch platforms were used for above-the-knee lesions, while 0.018-inch and 0.014-inch platforms were preferred for below-the-knee lesions. Switching to a lower-profile platform was at the operator’s discretion.

In cases where antegrade lesion crossing failed, retrograde pedal access was used for lesion crossing. After successful lesion crossing, balloon angioplasty was performed. Technical success was defined by <%30 residual stenosis after angioplasty, confirmed by control angiography. In cases of elastic recoil, flow-limiting dissection, or residual stenosis, bail-out stenting was performed. Drug-eluting balloons, atherectomy, and reentry devices were not used during the study period.

### 2.3. Use of iodine-based contrast medium

After placement of an appropriate vascular sheath proximal to the target lesion, diagnostic angiography was performed using a contrast volume range of 6–10 cc with a flow rate of 3–5 cc/s. Iohexol 350 mg/mL (Biemexol; Biem İlaç, İstanbul, Türkiye) was used as the contrast medium, diluted to a 50% mixture with 0.9% NaCl. Road mapping and blended road mapping were used when available to minimize contrast dose. During the intervention, hand injections were also used when needed, and the total amount of contrast used was recorded at the end of the procedure.

### 2.4. Use of CO_2_ during interventions

An automated CO_2_ delivery system (Angiodroid SRL, Bologna, Italy) was used during the interventions. The CO_2_ injector was connected to the side arm of the introducer sheath, and the CO_2_ volume was determined according to the region of interest. The treated limb was elevated up to 30 degrees for better visualization. An increased frame rate of 3–5 fps was used for image acquisition.

For imaging the above-the-knee segment, 20–30 cc of CO_2_ was used, while 10–30 cc was used for the below-the-knee segment. Injection pressure was set between 150–250 mmHg, typically adjusted to 20 mmHg above the patient’s systolic blood pressure. To prevent gas fragmentation and image degradation, the injection system was purged with 5 cc of gas prior to injections.

**Figure 2:**
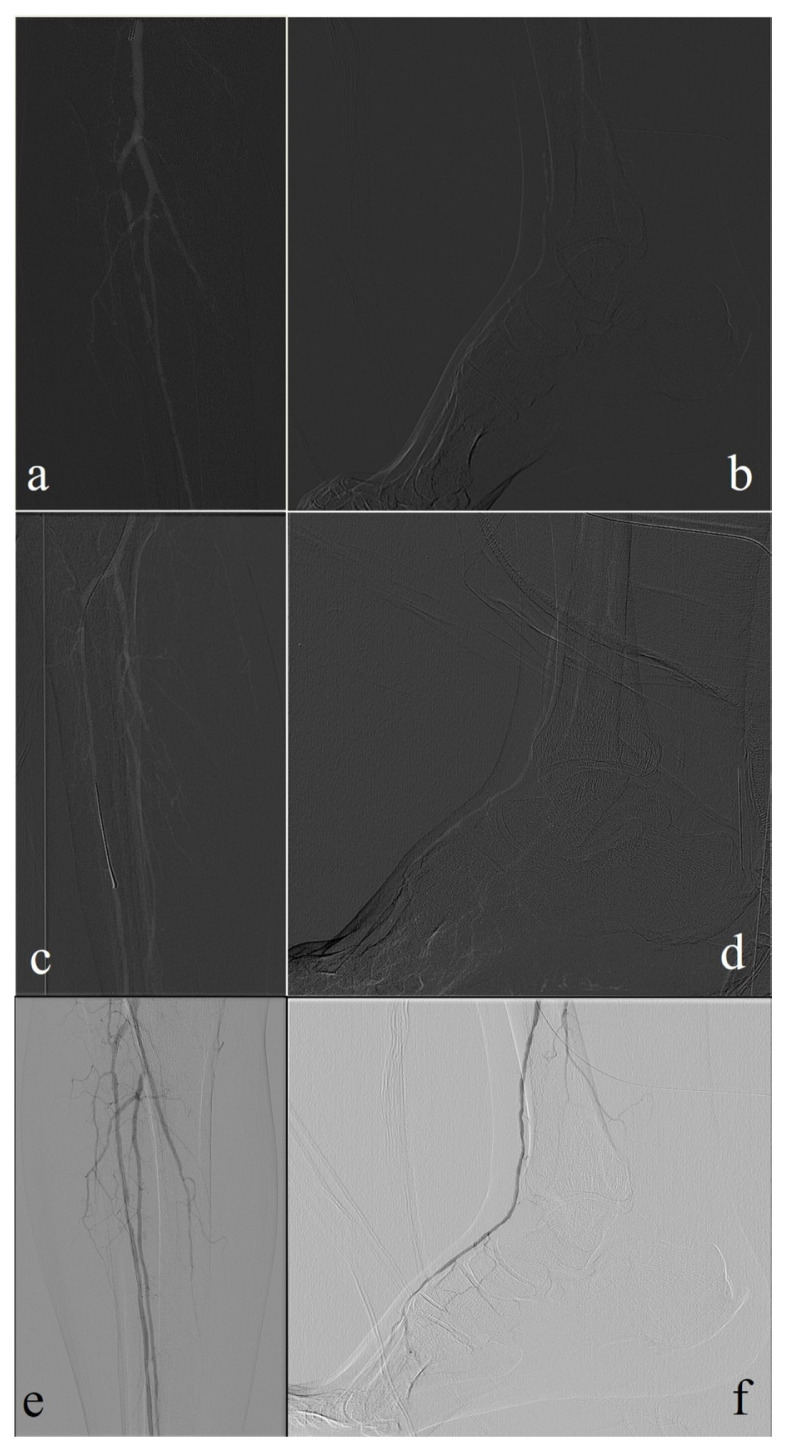
A 64-year-old patient with a foot ulcer involving the first toe. Diagnostic angiographies with CO_2_ reveal multilevel proximal stenosis and a long-segment occlusion of the anterior tibial artery (a), with distal recanalization (b). Final angiographic images with CO_2_ demonstrating successful recanalization of the anterior tibial artery (c). Image quality is reduced distally due to motion artifacts and contrast fragmentation (d). Control angiographies performed with diluted iodinated contrast media (e, f). A total of 12 cc of contrast was used during the procedure.

Image quality during CO_2_ angiography was assessed subjectively by the performing interventional radiologist in real-time. In cases of poor image quality, particularly in below-the-knee segments, supplemental iodinated contrast injections were administered to guide treatment as needed. However, these additional injections did not alter the overall treatment strategy. No objective image quality scoring system was employed, as image quality assessment is beyond the scope of the study. A sample case is presented in Figures 2a–2f.

### 2.5. Statistical analysis

All statistical analyses were performed using SPSS version 20 (IBM Corp., Armonk, NY, USA). Continuous variables were presented as mean ± standard deviation (SD) or median (minimum–maximum), depending on the distribution of the data. Categorical variables were presented as frequencies and percentages.

Kolmogorov–Smirnov test was used to assess the normality of continuous variables. Normally distributed continuous variables were compared using the Student t-test, while nonnormally distributed variables were compared using the Mann–Whitney U test. Categorical variables were compared using the Chi-square test or Fisher’s exact test, where appropriate. A p-value of <0.05 was considered statistically significant. Multivariate logistic regression analysis was performed to identify independent predictors of CIN. Odds ratios (OR) and 95% confidence intervals (CI) were reported. A p-value of <0.05 was considered statistically significant.

## 3. Results

The demographic characteristics of the patients were outlined in Table 1. Both groups were comparable in terms of age, sex, coronary artery disease, heart failure, anemia, smoking status, wound infection, Rutherford classification, and Wagner stages. All patients were diabetic, with similar glycemic control reflected by comparable hemoglobin A1c (HbA1c) levels.

Baseline serum creatinine levels were lower, and glomerular filtration rates (GFR) were higher in the control group. Chronic kidney disease (CKD) was significantly more common in the study group, and all patients in the study group had CKD stage 3 or higher.

The features of the interventions and clinical outcomes are summarized in Table 2. Both groups were comparable in terms of lesion level and total number of lesions treated. Technical success and complication rates were also similar between the groups.

The mean contrast volume used in the CO_2_ group was significantly lower than in the control group (24.3 ± 13.3 cc vs 89.4 ± 24.8 cc; p < 0.001). A total of 41 patients developed CIN. The incidence of CIN was 17.7% in the control group, while it was significantly lower in the CO_2_ group at 6.2% (p = 0.024). In the subgroup of patients with CKD stage 3–5 (n = 119), CIN occurred in 6.2% (4/64) of the CO_2_ group and 38.2% (21/55) of the contrast group (p < 0.001) (Table 3). The relative risk of CIN in the CO_2_ group was 0.16 (95% CI: 0.06–0.45), with an absolute risk difference of 31.9%. Multivariate logistic regression analysis demonstrated that CO_2_ angiography was independently associated with a significantly lower risk of CIN (OR: 0.027, 95% CI: 0.005–0.133, p < 0.001), even after adjusting for age, sex, comorbidities, CKD stage, Wagner classification, and HbA1c levels. CKD stage remained a strong independent predictor of CIN (OR: 2.69, p < 0.001) (Table 4).

Among patients who developed CIN, serum creatinine levels returned to baseline in 27 patients. However, renal function continued to deteriorate in 14 patients, and seven patients required hemodialysis. Three patients who progressed to end-stage renal disease died during their hospital stay.

In the study group, procedure times were recorded to be longer than those in the control group (57.2 min vs 49.2 min; p = 0.018). Technical success rates were comparable between the groups (93.7% vs 93.2%; p = 0.892). A total of 22 minor complications occurred, most of which were clinically subtle guidewire perforations and minor access-site hematomas. Among four patients who developed flow-limiting dissections, two required bail-out stenting. One case of distal embolization was successfully treated with catheter aspiration and full recanalization was achieved. No major complications related to the interventions were observed, and both groups had similar overall complication rates. No procedure-related mortality was observed.

Transient leg pain occurred in 11 patients following angiography, which was related to CO_2_ injection and resulted in image quality degradation due to involuntary limb movement. The pain resolved after administration of analgesics, and no further pain or involuntary movement was reported. There were no signs of nausea, vomiting, and mesenteric ischemia associated with CO_2_ use.

## 4. Discussion

This study demonstrated that the use of carbon dioxide (CO_2_) angiography effectively reduces both the contrast dose and the incidence of CIN following endovascular interventions in patients with diabetic foot ulcers. CIN is a major contributor to adverse cardiovascular events after endovascular interventions and is associated with prolonged hospitalization, increased healthcare costs, and higher mortality rates [11–14].

Although CIN after coronary interventions has been extensively studied, data regarding CIN following peripheral interventions is still evolving [15,16]. Reported CIN incidence after lower limb angioplasty varies widely, which can be attributed to differences in CIN definitions across studies and the heterogeneous characteristics of the included patient populations. Large studies conducted by Al Adas et al. and Grossmann et al. reported CIN rates of 6.5% and 3%, respectively [17,18]. However, these studies included both claudicant patients and those with critical limb ischemia (CLI), and not all patients were diabetic.

In the present study, the overall rate of CIN was 15.0%, which is higher than that reported in the aforementioned studies. This discrepancy may be due to the selection of a specific high-risk group of diabetic patients with foot ulceration. Diabetic patients are known to be at increased risk for CIN. Although the exact mechanisms are not fully understood, proposed contributing factors include renal hypoxia, increased oxidative stress, and tubular toxicity [4].

A higher CIN incidence of 19% was reported by Sigterman et al. [14] in patients with CLI undergoing endovascular interventions. Similarly, Cury et al. reported a CIN rate of 35.5% in CLI patients, supporting that patients with CLI are at significantly higher risk of CIN compared to claudicants [19].

Diabetic patients with foot ulcers are a specific population and differ from patients without wounds; they are more prone to CIN. Diabetic foot patients with Wagner stage 4 or more are reported to have more CIN than patients with lower stages [21,22]. Recurrent endovascular interventions, preexisting nephropathy, and the frequent use of analgesic drugs and antibiotics may also contribute to the elevated CIN prevalence in this group.

The high CIN rates observed in the present study emphasize that endovascular treatment in diabetic foot patients should be approached with the high risk of CIN in mind, even in the absence of preexisting renal disease.

Heart failure, elder age, hypertension, hyperlipidemia, anemia, and malnutrition are some of the other risk factors associated with CIN. Preexisting CKD and the amount of contrast media used are considered independent factors related to CIN [23–25]. Thus, minimizing contrast use is a critical aspect of prevention strategies. Although meticulous technique and the use of diluted iso-osmolar contrast agents can help reduce contrast volume to some extent, it is mostly not enough to achieve acceptable contrast doses per intervention.

In the current study, the mean contrast media volume used was significantly lower in the study group compared to the control group (24.3 cc vs. 89.4 cc). This finding supports the idea that CO_2_ angiography effectively limits contrast usage and decreases the risk of CIN. There were no patients with CKD stage 1 or stage 2 in the study group, which may be a confounding factor. To address the potential confounding effect of baseline renal function imbalance between groups, we conducted a dedicated subgroup analysis in patients with CKD stage 3–5. In this high-risk cohort, CIN incidence remained significantly lower in the CO_2_ group (6.2% vs. 38.2%, p < 0.001). Furthermore, multivariate logistic regression confirmed that CO_2_ use was an independent protective factor against CIN. These results strengthen the hypothesis that CO_2_ angiography has a genuine nephroprotective effect and that its benefit is not merely attributable to baseline risk differences.

There is still no clear consensus about contrast volume thresholds in endovascular interventions regarding the risk of CIN. In a prospective study, CIN risk was reported to increase with contrast volumes exceeding 50 cc during interventions, while in another study this limit was determined as 25 cc [5,21]. In a large-volume retrospective study, the recommended contrast volume thresholds were <50 cc for patients with CKD stage 3, <20 cc for CKD stage 4, and <9 cc for CKD stage 5 [20].

The literature on the use of CO_2_ angiography in peripheral endovascular interventions is still evolving. In a retrospective study, Stegemann et al. [8] compared CO_2_ angiography combined with iodinated contrast media (ICM) to angiography using ICM alone. The study included 191 consecutive patients and demonstrated that both contrast volume and CIN rates were significantly lower in the CO_2_ group. Unlike the current study, both claudicants and CLI patients were treated, and 51% of the patients were diabetic. In that study, CIN was only reported in CLI patients.

Another study involving 150 patients compared prospective data from 50 patients who underwent CO_2_ angiography to a matched retrospective cohort of 100 patients [5]. Lower rates of CIN were reported in the study group (14% vs. 29%), which was attributed to the significant reduction in mean contrast volume (15.1 cc vs 115.6 cc). Jakobi et al. also reported lower contrast volumes and reduced CIN rates in a prospective study involving patients with renal impairment (CKD stage 3 and higher). Their study further highlighted the increased risk of CIN in patients with both CLI and CKD stage 4–5 [21].

One of the largest studies investigating the efficacy of CO_2_ angiography, conducted by Lee et al., demonstrated that CO_2_ angiography reduces the ICM volume and is associated with lower rates of cardiac complications and CIN [3]. In contrast to these findings, Locham et al. studied the role of prophylactic intravenous hydration and CO_2_ angiography in high-risk CKD patients. Their study concluded that both intravenous hydration and CO_2_ angiography failed to reduce contrast-associated acute kidney injury [26]. Mean contrast volumes used in the study were 66.89 cc in the CO_2_ group, compared to 65.94 cc in the control group, which may largely explain the lack of benefit observed. These discrepancies point out the need for prospective, multicenter, controlled trials to further evaluate the role of CO_2_ angiography in high-risk patients undergoing endovascular interventions for lower limb disease.

The main challenge of CO_2_ angiography in the endovascular treatment of CLI is achieving high-quality images. Pain during CO_2_ injection and associated limb movement are common causes of image degradation. Additionally, fragmentation of CO_2_ during injection is another reason for low image quality. This issue is more pronounced in the below-the-knee segment [7,9,27]. Low-quality images mandate the use of ICM and increase the risk of CIN indirectly. In this study, an automated CO_2_ delivery system was used to obtain optimal image quality during interventions. Palena et al. reported an overall diagnostic accuracy of 89.8% (sensitivity 92.3% and specificity 75.0%) in a prospective study with 36 patients. However, in the below-knee and below-ankle segments, sensitivity rates were 93.3% and 86.6%, and specificity rates were 83.3% and 66.6%, respectively [7].

In another prospective study on 100 patients using CO_2_ angiography, poor image quality was reported in 82.6% of the below-knee arteries. The image quality of femoropopliteal lesions was shown to be comparable to conventional angiography in a randomized single-center prospective study [28].

In a recent prospective multicenter observational study over 114 CLI patients who underwent infrainguinal endovascular intervention, no cases of CIN were observed [27]. As imaging is the key step of endovascular intervention and most diabetic patients are affected in the below-the-knee segment, the quality of imaging is a major concern. Standardization of the technique and improvement of imaging quality will be topics for future studies.

The retrospective and single-center design of this study constitutes a major limitation. Due to the nonrandomized nature of the analysis, selection bias could not be entirely excluded. Additionally, during the study period, some of the patients underwent a one-day procedure and due to lack of follow-up data, these patients were excluded from the study. As a result, exact CIN incidence may be underreported in our study. The lack of a long-term follow-up protocol, due to the retrospective nature of the study, limits the generalization of our data regarding the long-term nephrotoxicity of contrast media. Although the study and control groups were matched for most baseline characteristics, CKD stages and serum creatinine levels were higher in the study group. On the other hand, this study showed that even with normal serum creatinine levels and GFR, diabetic foot patients are under substantial risk of CIN. Another limitation is that only infrainguinal procedures were included in the study due to the common distribution of the disease at this anatomic level. However, aortoiliac disease should not be overlooked, as concomitant aortoiliac interventions may be needed for treatment and may require high volumes of contrast media. These limitations restrict the generalizability of the findings and highlight the need for future randomized, multicenter, prospective trials to confirm the nephroprotective role of CO_2_ angiography in high-risk populations.

In conclusion, this study demonstrated that CO_2_ angiography effectively reduces the contrast dose during lower limb interventions and the incidence of postintervention CIN. These findings contribute to the growing body of literature on CIN in diabetic patients with foot ulcers. CO_2_ angiography may play a crucial role in minimizing the risk of CIN in this specific population, who are more vulnerable to this complication and its associated morbidity and mortality. Further multicenter, prospective studies are needed to better define the role of CO_2_ angiography in high-risk patients and to establish standardized protocols for its use.

## Figures and Tables

**Table 1:** Demographic characteristics of the patients.

	Study group	Control group	p-value
**Age (mean) (SD)**	67.81 (8.5)	65.5 (9.9)	0.893
**Male sex (n/total)**	48/64	152/208	0.760
**CAD (n/total)**	38/64	116/208	0.611
**HF (n/total)**	19/64	61/208	0.956
**Anemia (n/total)**	32/64	102/208	0.283
**Hypertension (n/total)**	23/64	123/208	0.101
**Dyslipidemia (n/total)**	41/64	109/208	0.442
**Smoking (n/total)**	29/64	83/208	0.157
**CKD stage (n)**			<0.001
**1**	0	46	
**2**	0	107	
**3a**	3	40	
**3b**	39	9	
**4**	17	4	
**5**	5	2	
**Wagner stage (n)**			0.079
**1**	3	36	
**2**	14	28	
**3**	11	48	
**4**	33	90	
**5**	3	6	
**Rutherford stage (n)**			0.859
**5**	58	190	
**6**	6	18	
**Wound infection (n)**	25	86	0.069
**HbA1c (mean) (SD)**	8.5 (1.9)	8.1 (1.5)	0.217
**Serum creatinine (mean) (SD)**	2.21 (0.84)	1.96 (0.63)	<0.001
**BUN (mean) (SD)**	68.0 (35.6)	43.4 (16.3)	<0.001
**GFR (mean) (SD)**	32.5 (10.6)	71.4 (20.1)	<0.001

* CAD: Coronary artery disease; HF: heart failure; CKD: chronic kidney disease; BUN: blood urea nitrogen; GFR: glomerular filtration rate; n: number; SD: standard deviation.

**Table 2:** Features of the interventions and clinical outcomes.

		Study group	Control group	p-value
**Lesion level**				0.227
** ** **Femoropopliteal (n)**		6	14	
** ** **Below the knee (n)**		30	99	
** ** **Combined (n)**	***FP+BTK***	4	38	
	***BTK+BTA***	20	45	
	***FP+BTK+BTA***	4	12	
**Total lesions treated (median) (min–max)**		2 (1–5)	2 (1–5)	0.772
**Mean procedure time (minutes) (min–max)**		57.2 (21–167)	49.2 (23–97)	0.018
**Mean contrast dose (cc) (SD)**		24.3 (13.3)	89.4 (24.8)	<0.001
**Technical success (%)**		93.7	93.2	0.892
**Complications (n)**	***Access site hematoma***	3	5	0.274
	***Thromboembolic***	1	0	
	***Dissection***	0	4	
	***Perforation***	2	7	
**CIN (n/total); (%)**		4/64; 6.2	37/208; 17.7	0.024

* FP: femoropopliteal; BTK: below-the-knee; BTA: below-the-ankle; CIN: contrast-induced nephropathy; SD: standard deviation; min–max: minimum-maximum; n: number.

**Table 3:** CIN incidence in patients with CKD Stage 3a–5.

CKD stage 3a–5 patients	CO_2_ group (n=64)	Contrast group (n=55)	p-value
**CIN (+), n (%)**	4 (6.2%)	21 (38.2%)	<0.001
**CIN (**−**), n (%)**	60 (93.8%)	34 (61.8%)	

* CKD: chronic kidney disease; CIN: contrast-induced nephropathy; n: number.

**Table 4:** Predictors of contrast-induced nephropathy.

	OR (Exp(β))	95% CI	p-value
CO_2_ use	0.027	0.005–0.133	<0.001
CKD stage	2.70	1.73–4.20	<0.001
Heart failure	2.16	0.95–4.89	0.066
Hypertension	2.30	0.98–5.38	0.056
Wagner stage 4–5	1.76	0.81–3.82	0.155
Age	1.01	0.97–1.05	0.717
HbA1c	1.00	0.81–1.23	0.941

* CO_2_: carbon dioxide;

CKD: chronic kidney disease; CI: confidence interval; OR: odds ratio.
