# Erratum to “ The role of carbon dioxide angiography in reducing contrast-induced nephropathy in diabetic foot patients undergoing endovascular treatment” [Turkish Journal of Medical Sciences 55 (4): 877–886]

**DOI:** 10.55730/1300-0144.6091

**Published:** 2025-10-14

**Authors:** Sadık Ahmet UYANIK, Erdem BİRGİ, Saffet ÖZTÜRK, Umut ASFUROĞLU, Erdi TANGOBAY, Hikmet Erhan GÜVEN

**Affiliations:** 1 Department of Radiology, Etlik City Hospital, Ankara, Turkiye; 2 Department of Radiology, Ankara Training and Research Hospital, University of Health Sciences, Ankara, Turkiye; 3 Department of General Surgery, Etlik City Hospital, Ankara, Turkiye

In Table 1 of this article, the baseline serum creatinine value for the control group was incorrectly reported due to typing error.

- Incorrect value (published): 1.96 ± 0.63 mg/dL
- Correct value: 1.06 ± 0.63 mg/dL

This correction does not affect the statistical analyses, results, or conclusions of the study. The authors apologize for this error and any confusion it may have caused.

**Table 1:** Demographic characteristics of the patients

	Study group	Control group	P value
**Age (mean) (SD)**	67.81 (8.5)	65.5 (9.9)	0.893
**Male sex (n/total)**	48/64	152/208	0.760
**CAD (n/total)**	38/64	116/208	0.611
**HF (n/total)**	19/64	61/208	0.956
**Anemia (n/total)**	32/64	102/208	0.283
**Hypertension (n/total)**	23/64	123/208	0.101
**Dyslipidemia (n/total)**	41/64	109/208	0.442
**Smoking (n/total)**	29/64	83/208	0.157
**CKD stage (n)**			<0.001
**1**	0	46	
**2**	0	107	
**3a**	3	40	
**3b**	39	9	
**4**	17	4	
**5**	5	2	
**Wagner stage (n)**			0.079
**1**	3	36	
**2**	14	28	
**3**	11	48	
**4**	33	90	
**5**	3	6	
**Rutherford stage (n)**			0.859
**5**	58	190	
**6**	6	18	
**Wound infection (n)**	25	86	0.069
**HbA1c (%) (mean) (SD)**	8.5 (1.9)	8.1 (1.5)	0.217
**Serum creatinine (mg/dL) (mean) (SD)**	2.21 (0.84)	1.06 (0.63)	<0.001
**BUN (mg/dL) (mean) (SD)**	68.0 (35.6)	43.4 (16.3)	<0.001
**GFR (mL/min/1.73m** **^2^** **) (mean) (SD)**	32.5 (10.6)	71.4 (20.1)	<0.001

* CAD: Coronary artery disease; HF: heart failure; CKD: chronic kidney disease; BUN: blood urea nitrogen; GFR: glomerular filtration rate; n: number; SD: standard deviation

